# Angiogenesis and immune checkpoint inhibitors as therapies for hepatocellular carcinoma: current knowledge and future research directions

**DOI:** 10.1186/s40425-019-0824-5

**Published:** 2019-11-29

**Authors:** Marc Hilmi, Cindy Neuzillet, Julien Calderaro, Fouad Lafdil, Jean-Michel Pawlotsky, Benoit Rousseau

**Affiliations:** 1 Department of Medical Oncology, Curie Institute, University of Versailles Saint-Quentin, Paris, France; 2 Department of Pathology, Henri Mondor Hospital, Créteil, France; 3 IMRB-INSERM U955 Team 18, Créteil, France; 4 Université Paris-Est-Créteil, Créteil, France; 5 Institut Universitaire de France, Paris, France; 6 National Reference Center for Viral Hepatitis B, C and D, Department of Virology, Henri Mondor Hospital, Créteil, France; 7 Department of Medicine, Division of Solid Tumor Oncology, Memorial Sloan Kettering Cancer Center, New York, NY USA

**Keywords:** Hepatocellular carcinoma, Checkpoint inhibitor, Drug combination, Immunology, Tumor microenvironment

## Abstract

Hepatocellular carcinoma (HCC) is the second deadliest cancer worldwide, due to its high incidence and poor prognosis. Frequent initial presentation at advanced stages along with impaired liver function limit the use of a broad therapeutic arsenal in patients with HCC. Although main HCC oncogenic drivers have been deciphered in recent years (*TERT, TP53, CTNNB1 mutations, miR122 and CDKN2A silencing*), therapeutic applications derived from this molecular knowledge are still limited. Given its high vascularization and immunogenicity, antiangiogenics and immune checkpoint inhibitors (ICI), respectively, are two therapeutic approaches that have shown efficacy in HCC. Depending on HCC immune profile, combinations of these therapies aim to modify the protumoral/antitumoral immune balance, and to reactivate and favor the intratumoral trafficking of cytotoxic T cells. Combination therapies involving antiangiogenics and ICI may be synergistic, because vascular endothelial growth factor A inhibition increases intratumoral infiltration and survival of cytotoxic T lymphocytes and decreases regulatory T lymphocyte recruitment, resulting in a more favorable immune microenvironment for ICI antitumoral activity. First results from clinical trials evaluating combinations of these therapies are encouraging with response rates never observed before in patients with HCC. A better understanding of the balance and interactions between protumoral and antitumoral immune cells will help to ensure the success of future therapeutic trials. Here, we present an overview of the current state of clinical development of antitumoral therapies in HCC and the biological rationale for their use. Moreover, translational studies on tumor tissue and blood, prior to and during treatment, will help to identify biomarkers and immune signatures with predictive value for both clinical outcome and response to combination therapies.

## Introduction

Hepatocellular carcinoma (HCC) is the most frequent primary liver cancer and the second leading cause of cancer death worldwide [1]. Despite significant progress in the diagnosis and treatment of HCC, its prognosis remains extremely poor with a 5-year overall survival (OS) rate of 12%, all stages taken together [1]. Most HCCs (80–90%) develop on underlying chronic liver disease (with or without cirrhosis); the main causes include chronic hepatitis B virus (HBV) or hepatitis C virus (HCV) infections, alcohol consumption, non-alcoholic steatohepatitis, or other less frequent etiologies such as hemochromatosis, tobacco and aflatoxin B1 [2–6]. The highest incidence of HCC is observed in South-East Asia and Central Africa, where the endemic prevalence of chronic HBV infections accounts for 70% of cases [7, 8].

**Fig. 1:**
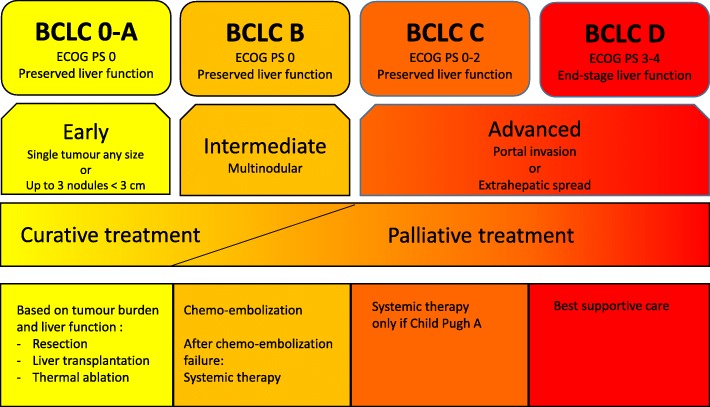
“Barcelona-Clinic Liver Cancer” (BCLC) classification and treatment of hepatocellular carcinoma according to the 2018 ESMO and EASL Clinical Practical Guidelines. ECOG PS: Eastern Collaborative Oncology Group Performance Status; TACE: transarterial chemoembolization

The “Barcelona Clinic Liver Cancer” (BCLC) classification is currently recommended to assess the prognosis and choose the most appropriate treatment for HCC patients [8–12] (Fig. 1, available online at https://www.esmo.org/Guidelines/Gastrointestinal-Cancers/Hepatocellular-Carcinoma). There are five BCLC classes (0, A, B, C and D) which take into consideration both the underlying liver function, as assessed by the Child-Pugh score, and the patient’s general condition according to the Eastern Collaborative Oncology Group Performance Status (ECOG PS). The only curative treatments for HCC, reserved to patients with early-stage HCC (BCLC stage 0, A), are surgical resection, thermal ablation, radiotherapy and/or liver transplantation [8, 9, 11, 12]. No adjuvant treatment has been validated for HCC.

In the more than 70% of patients diagnosed with unresectable HCC (at the intermediate BCLC B stage or at the advanced C-D stages), treatments can only be palliative [8, 9, 12]. HCC is one of the most chemoresistant tumor, and the use of cytotoxic agents is frequently limited by the altered underlying liver function that increases their toxicity [7, 8]. Hence, doxorubicin and PIAF (platinum, interferon, doxorubicin and 5-fluoropyrimidine) combinations, tested in phase III trials in advanced HCC, did not show any survival benefit [7, 8, 12, 13]. A trend towards improved OS was observed with the FOLFOX regimen (5-fluoropyrimidine, leucovorin, and oxaliplatin) as compared to doxorubicin [14], and with the GEMOX regimen (gemcitabine, oxaliplatin) [15]. However, cytotoxic chemotherapy is not recommended at advanced stages of HCC, and should remain a therapeutic option only in patients who cannot receive standard treatment [12].

Antiangiogenics and immunotherapies represent the main avenues in the treatment of advanced HCC. The objective of this review is to provide an overview of current clinical development of these therapies alone or in combination in patients HCC and discuss the biological rationale for their use according to the underlying intratumoral immune profiles.

## Targeted therapies

### Rationale for angiogenesis inhibitors

Many proangiogenic growth factors are overexpressed in HCC, such as vascular endothelial growth factor A (VEGFA), platelet-derived growth factor (PDGF), IGF-1 and TGF-β [8, 16]. *VEGFA* gene amplifications have been described in 4 to 8% of HCCs, thereby inducing both neoangiogenesis and tumor proliferation via the induction of hepatocyte growth factor secretion by macrophages [17]. Overall, HCCs are highly vascularized tumors with predominant arterial blood flow, making them good candidates for both antiangiogenic agents and arterial endovascular procedures, such as chemoembolization.

### Clinical applications (Table 1)

#### Sorafenib: clinical development

In 2008, sorafenib became the first systemic treatment to demonstrate a significant survival benefit in patients with advanced HCC. Sorafenib is a multikinase inhibitor (MKI) that reduces both HCC cell proliferation and angiogenesis by targeting a broad spectrum of protein kinases, including VEGFR, PDGFR, c-KIT and RAF. Two phase 3 trials (SHARP and ASIA-PACIFIC) evaluating sorafenib versus placebo showed a significant increase in median OS in patients with preserved liver function (Child-Pugh A) and advanced HCC (BCLC C or BCLC B with tumor progression after locoregional therapy and naive of systemic therapy) [18, 19]. Diarrhea, hand-foot syndrome, and fatigue were the most frequent adverse events, causing approximately 8% of grade 3–4 events each. Exploratory subgroup analyses of the SHARP study showed that sorafenib increased OS and disease control rate (DCR) relative to placebo regardless of etiology, initial tumor volume, ECOG PS, and previous treatments [23]. The ASIA-PACIFIC study was a mirror clinical trial of the SHARP study in a population of Asian patients [19]. The shorter OS (6.5 versus 4.2 months) observed in the ASIA-PACIFIC study may be explained by the higher frequency of poor prognostic factors in the patients included, with large tumor volumes, high prevalence of HBV infection, and altered ECOG PS [24].

**Table 1:** Summary of positive phase 3 clinical trials of angiogenic inhibitors in patients with advanced hepatocellular carcinoma (HCC)

*Molecules*	*Trial*	*N*	*Population*	*mOS*	*mPFS*	*ORR*	*DCR*	*Comments*
Sorafenib	SHARP [18]	602	First-line Versus placebo	10.7 m	5.5 m	2%	43%	
	ASIAPACIFIC [19]	226	First-line Versus placebo	6.5 m	2.8 m	3.3%	35.3%	
Lenvatinib	REFLECT [20]	954	First-line Versus sorafenib	13.6 m	8.9 m	24.1, 1%CR	75.5%	
Regorafenib	RESORCE [21]	573	Second-line Versus placebo	10.6 m	3.2 m	11%	65%	Exclusion of patients previously intolerant to sorafenib
Cabozantinib	CELESTIAL [22]	707	Second or third-line Versus placebo	10.2 m	5.2 m	4%	64%	Inclusion of patients previously intolerant to sorafenib
Ramucirumab	REACH-2 (35)	292	Second-line Versus placebo	8.5 m	2.8 m	4.6%	59.9%	Inclusion of patients with poor prognosis based on high alpha-foeto-protein levels

*CR* complete response; *DCR* disease control rate; *m* months; *mOS* median overall survival; *mPFS* median progression-free-survival; *N* number of randomized patients; *ORR* objective response rate

Following these two pivotal trials, sorafenib obtained worldwide approval and became the standard first-line treatment for advanced HCC. No predictive markers of response had been identified in the translational studies derived from the SHARP study [25]. Since then, several predictive biomarkers have been proposed, including amplifications of fibroblast growth factor 3/4 or VEGF-A, polymorphisms of VEGF-A and VEGF-C, or tissue expression of pERK or VEGFR-2 [17] and imaging criteria [26]. However, none of these biomarkers has been validated for clinical use with antiangiogenics. Combinations of sorafenib with erlotinib [27], doxorubicin [28] or transarterial chemoembolization [29] has been explored in randomized trials, without improvement of OS or progression-free survival (PFS) [27, 28]. The reasons for these failures were limiting toxicities and the absence of patient selection based on molecular markers.

#### Other first-line therapies

Since the approval of sorafenib, new candidate drugs failed to demonstrate their efficacy as first-line therapies versus sorafenib: they included sunitinib [30], brivanib [31] and linifanib [32]). In 2018, a non-inferiority trial evaluating lenvatinib versus sorafenib was published [20]. Lenvatinib is an angiogenesis inhibitor targeting multiple tyrosine kinase receptors, including VEGF receptors 1 to 3, FGF receptors 1 to 4, PDGF receptor, RET and KIT. This non-inferiority trial in patients with BCLC B or C HCC and Child-Pugh A showed similar efficacy of lenvatinib and sorafenib in terms of median OS (13.6 months versus 12.3 months, respectively), with improved median PFS (7.4 months versus 3.7 months, respectively) and objective response rate (ORR) according to modified RECIST criteria (24% versus 9%, respectively). In addition, the toxicity profile of lenvatinib was more favorable than that of sorafenib (lower incidence of fatigue, diarrhea and hand-foot syndromes). Together, these results led to lenvatinib approval by the Food and Drug Administration.

#### Second-line therapies and beyond

Several drugs have failed versus placebo in second-line treatment trials after failure of or intolerance to sorafenib, including brivanib [33] or everolimus [34]. In 2016, the RESORCE phase 3 trial showed that regorafenib, a sorafenib derivative whose structure differs by the addition of a fluorine atom, significantly improved median OS by 3 months, as compared to placebo, as second-line treatment after failure of sorafenib to prevent disease progression (hazard ratio (HR) = 0.63; *p* < 0.0001, 33). The most frequent grade 3–4 adverse events on regorafenib were hypertension (15%), hand-foot syndrome (13%), fatigue (9%) and diarrhea (3%).

The CELESTIAL phase 3 trial subsequently evaluated cabozantinib, an MKI targeting VEGFR 1 to 3, c-MET and AXL, all involved in sorafenib resistance, as second- or third-line therapy in patients previously treated with sorafenib [22]. The study showed a 2-month benefit for median OS in favor of cabozantinib, as compared to placebo (HR = 0.76; *p* = 0.005). The most common serious adverse events were hand-foot syndrome (17%), hypertension (16%), liver function disorders (12%), fatigue (10%) and diarrhea (10%).

Finally, the REACH-2 phase 3 trial evaluated ramucirumab, an anti-VEGFR-2 monoclonal antibody in patients with advanced HCC pre-treated with sorafenib and with a high alpha-fetoprotein (AFP) level (≥ 400 ng/mL) [35]. This study was designed following the results of the REACH-1 trial; in this phase 2 study, the primary objective was not met in the overall HCC patient population (unselected for AFP), but a benefit was suggested in the subgroup of patients with elevated AFP at the initiation of treatment [36]. REACH-2 showed a modest but significant survival benefit, as compared to placebo: 8.5 months versus 7.3 months, respectively (HR = 0.71; *p* = 0.019). Thus, ramucirumab is the first biomarker-guided therapy to show efficacy in patients with HCC. Due to the lack of liver metabolism, ramucirumab had a milder toxicity profile, as compared to MKI, inducing mainly hypertension (12% of grade ≥ 3) and hyponatremia (5.6% of grade ≥ 3).

In summary, cabozantinib, regorafenib, and ramucirumab have recently become new therapeutic options in patients with advanced HCC previously treated with sorafenib. Interestingly, the efficacy of these three drugs is within the same range, with a 25% reduction of the risk of death, albeit in non-comparable patient populations. Indeed, REACH-2 included patients with a poorer prognosis than the other trials, due to the selection based on high AFP levels, which may explain the lower survival rate observed in this study. Finally, no biomarker has been identified to guide the choice between these three angiogenesis inhibitors in clinical practice. Furthermore, whether a treatment sequence effect similar to angiogenic blockade beyond progression in colorectal cancer (with angiogenesis being continuously blocked) exists in HCC remains unknown.

### Perspectives: molecular alterations in HCC

Hepatocarcinogenesis is a complex multi-step process in which many signaling pathways are altered. The understanding of molecular pathogenesis of HCC has deeply improved over the last decade [37]. Genomic analyses, mainly based on the study of resected tumor samples, provided an overview of biological drivers responsible for the initiation and progression of HCC. The most frequent mutations involve: (i) telomere maintenance (mutations in the telomerase reverse transcriptase (*TERT*) promoter); (ii) the WNT-β catenin pathway (*CTNNB1*/β-catenin activating mutations); (iii) p53 tumor suppressor (inactivating mutations of *TP53*) and cell cycle control; (iv) chromatin remodeling and other epigenetic modifiers (mutations in AT-rich interaction domain 1A[*ARID1A*]); (v) MAP kinases and mechanistic target of rapamycin signaling pathways; and (vi) oxidative stress pathways [8, 38]. Activations of c-MET, insulin-like growth factor (IGF) receptor, fibroblast growth factor receptor (*FGF19* amplification), epidermal growth factor receptor, Hedgehog, JAK/STAT and transforming growth factor β (TGF-β) signaling have also been described [39]. In order to offer targeted treatments to patients, i.e. treatments adapted to their molecular profile, it has been proposed to define HCC subgroups with homogeneous oncogenic alteration profiles.

In 2015, a first molecular classification divided HCC into two main classes, each representing about 50% of patients, including [38]: (i) the proliferative class, enriched in activation of the RAS pathway, mechanistic target of rapamycin and IGF signaling pathways, *FGF19* amplification, associated with HBV infection and with a poor prognosis; (ii) the non-proliferative class, more heterogeneous but characterized by *CTNNB1* mutations and associated with alcohol and HCV infection.

In 2017, the international consortium “The Cancer Genome Atlas (TCGA) Research Network” proposed a new classification based on the cross-platform analysis of 363 cases of HCC by whole-exome sequencing and DNA copy number analysis, and the additional analysis of 196 cases for DNA methylation, RNA expression, miRNA, and proteomics [40]. The authors identified three integrated clusters (iClust) of HCC. Main molecular alterations from TCGA are presented in Table 2. iClust 1 was associated with earlier age, Asian origin and female gender. It was dominated by high-grade tumors, with macrovascular invasion and overexpression of proliferation markers. At the molecular level, iClust 1 had a low mutation frequency of *CTNNB1* (12%), epigenetic silencing of *CDKN2A* (32%), and a low expression of TERT, as compared to other clusters. Patients with iClust 1 tumors had the worst prognosis. iClust 2 and iClust 3 had a high frequency of *CDKN2A* silencing by hypermethylation, *TERT* promoter mutations, *CTNNB1* mutations, and enrichment in *HNF1A* mutations. iClust 2 was significantly associated with a low-grade tumor phenotype and limited microvascular invasion. iClust 3 was associated with high chromosomal instability including 17p loss, high frequency of *TP53* mutations, and hypomethylation of multiple CpG sites. This study also revealed new alterations in iClust 3, such as mutations in isocitrate dehydrogenase genes (*IDH1/2*).

**Table 2:** Main molecular alterations in HCC according to molecular subtypes adapted from the Cancer Genome Atlas Research Network [40]

Molecular subtype	Genetic features	Epigenetic features	Other characteristics
iCluster 1 36%	Few *CTNNB1* mutations Low expression of TERT Overexpression of proliferation markers	microRNA signature mir-122 silencing High expression of miR-181A	Young age Asian patients Female patients Normal Weight High grade Poor prognosis
iCluster 2 30%	High expression of TERT High expression of CTNNB1 *HNF1A* mutations	*CDKN2A* silencing	Low grade Low microvascular invasion
iCluster 3 34%	Chromosomal instability 17p loss *TP53* mutations High activation of CTNNB1 High expression of TERT Activation of VEGF-A pathway *PTEN* inactivation	*CDKN2A* silencing CPG Island hypomethylation	–
Frequencies of most prevalent alterations in the whole cohort	TERT mutations 44% TP53 mutations 31% CTNNB1 mutations 27% CDKN2A deletion 13% APOB mutations 10% AXIN1 mutations 8% ARID1A mutations 7%	*CDKN2A silencing 54%*	–

Recently, a prospective genotyping study in 81 patients with advanced HCC treated with sorafenib showed that mechanistic target of rapamycin (mTOR) signaling pathway alterations were associated with a poorer DCR (8.3% versus 40.2% in patients without such alterations) and OS (10.4 versus 17.9 months, respectively) [41]. Despite these advances in the understanding of oncogenic drivers of HCC, only few of them have been identified as actionable targets for therapy. Thus, these discoveries have not yet made it possible to offer personalized HCC management in clinical practice [42].

## Immune therapies

### Liver as an immune organ

The liver receives blood flow through both the portal vein and the hepatic artery and hosts a broad variety of innate and adaptive immune cells. The liver is responsible for the production of many pro-inflammatory cytokines and proteins. It is classically considered as a first-line immunological organ that plays a key role in the defense against infections of blood and digestive origin [43, 44]. Because of its anatomical location, the liver is perpetually exposed to pathogens and exogenous non-pathogenic molecules. Thus, the balance between immune activation and tolerance is finely regulated, in order to prevent an inadequate immune response against exogenous antigens from food and microbiota [43].

The existence of a link between antitumor immunity and HCC is supported by the observation of spontaneous tumor regressions upon discontinuation of immunosuppressive treatments in patients with liver transplantations [45]. Immunity-modulating therapies have been and are studied in numerous clinical trials in patients with HCC. Among these, immune checkpoint inhibitors (ICI) targeting the programmed cell death-1 (PD-1) axis are currently being studied as monotherapies or in combination with other ICI, such as cytotoxic T lymphocyte antigen-4 (CTLA-4) or angiogenesis inhibitors. CTLA-4 blockade affects the immune priming phase occurring in the lymph node and reduces regulatory T lymphocytes (Treg)-mediated suppression of effector T cells, while PD-1 blockade affects the effector phase in the tumor and restores the immune function of “exhausted” T cells.

### Clinical applications (Table 3)

#### Anti-CTLA-4

Two trials evaluated the safety and efficacy of tremelimumab, a monoclonal antibody inhibiting CTLA-4, alone or in combination with ablation therapy (chemoembolization or radiofrequency), respectively [46, 47]. The first study was a phase 2 trial evaluating tremelimumab in 20 patients with advanced HCC and chronic HCV infection [46]. These patients were heavily pretreated, with non-resectable or metastatic HCC (BCLC C: 57%, portal vein invasion: 28%), high tumor volume, and frequent altered liver function (Child-Pugh B: 43%). Tremelimumab resulted in a partial response rate of 17.6% and a DCR of 76.4%. The second study combined tremelimumab with ablation therapy in patients with advanced HCC in order to induce tumor necrosis, thereby promoting the release of tumor antigens to increase the efficacy of anti-CTLA-4 [47]. Thirty-two patients were included, but only 19 were radiologically evaluable. Patients with radiological benefit (26.3% ORR, 63% DCR) had an increase in intratumoral CD8+ T cells on biopsies performed 6 weeks after the start of treatment. In both studies, antitumoral activity and antiviral activity against HCV (decreased viral load) were observed and tolerance was acceptable without dose-limiting toxicities.

**Table 3:** Summary of clinical trials of immune therapies (single agent or combination with angiogenics inhibitors) in patients with advanced hepatocellular carcinoma (HCC)

*Type of immunotherapy*	*Molecules*	*Trial*	*Phase*	*N*	*Population*	*mOS*	*mPFS*	*ORR*	*DCR*
Anti-CTLA-4	Tremelimumab	Sangro et al. [46]	II	20	Pre-treated	8.2 m	6.5 m	17.6%	76.4%
		Duffy et al. [47]	II	32	Pre-treated Combination with ablation	12.3 m	7.4 m	26.3%	63%
Anti-PD-1	Pembrolizumab	Zhu et al. [48]	II	104	Pre-treated	12.9 m	4.9 m	17, 1% CR	60%
		Finn et al. [49]	III	413	Pre-treated	13.9 m	2.8 m	18%	NA
	Nivolumab	El-Kouheiry et al. [50]	I/II	262	Pre-treated and naive	NR	4 m	20, 1% CR	64%
	Cemiplimab	Pishvaian et al. [51]	I	26	Pre-treated	NR	3.7 m	19.2%	73%
Anti-PD-L1	Durvalumab	Wainberg et al. [52]	I/II	39	Pre-treated	13.2 m	NA	10.3%	33% at 24 weeks
Combinations									
Anti-PD-1 + Anti CTLA-4	Nivolumab + ipilumumab	Yau et al. [53]	II	148	Pre-treated	24-m OS 40%	NA	31, 5% CR	49%
Angiogenesis and immune checkpoints inhibitors	Atezolizumab + bevacizumab	Pishvaian et al. [54]	Ib	68	Naive	NR	14.9 m	34, 1% CR	78%
	Pembrolizumab + lenvatinib	Ikeda et al. [55]	Ib	18	Naive	NA	NA	46%	92%
	Camrelizumab + apatinib	Xu et al. [56]	I	16	Pre-treated	NR	5.8 m	50%	93.8%
	Avelumab + axitinib	Kudo et al. [57]	Ib	22	Naive	NR	5.5 m	13.6%/31.8% (mRECIST)	NA
Cytotoxic agents and Anti-PD-1	FOLFOX4 or GEMOX + camrelizumab	Qin et al. [58]	II	34	Naive	NR	5.5 m	26.5%	79.4%

*CR* complete response; *CTLA-4* Cytotoxic T lymphocyte-associated protein 4; *DCR* disease control rate; *m* months; *mOS* median overall survival; *mPFS* median progression-free-survival; *N* number of randomized patients; *NR* not reached; *NA* not available; *ORR* objective response rate; *PD-1*programmed cell death-1; *PD-L1* Programmed death-ligand 1

The results of these studies were encouraging but should be analyzed with caution, due to the limited sample size with a large proportion of patients not reaching the first radiological evaluation due to early clinical progression. In addition, industrial developments of anti-CTLA-4 now focuses on associations with anti-PD-1/programmed cell death-ligand 1 (PD-L1) antibodies in advanced stage HCCs (NCT03298451, [59]) or in the neo-adjuvant setting (NCT03510871). These combination studies rapidly emerged in the context of data showing the activity of anti-PD-1/PD-L1 monotherapy in advanced HCC, supported by a more favorable toxicity profile than anti-CTLA-4.

#### Anti-PD-1/PD-L1

While PD-1 receptor is mainly expressed by activated lymphocytes, PD-L1 ligand has been identified at the surface of tumor cells, as well as in the peritumoral stroma of HCCs and its presence is a poor prognostic factor [60]. In a cohort of 217 resected HCCs [61], PD-L1 expression within HCC tumors was found in about 75% of cases, with a wide range of intensity [61]. It has been suggested that PD-L1 expression should be assessed: (i) on tumor cells (threshold of 1%), and (ii) on immune cell clusters infiltrating the tumor [61]. High PD-L1 expression was associated with markers of tumor aggressiveness (high AFP levels, satellite nodules, poor differentiation, macro- and micro-vascular invasion). These observations support the potential therapeutic interest of blocking the PD-1/PD-L1 axis in HCC.

The first results of studies evaluating anti-PD-1/PD-L1 monotherapies as post-sorafenib second-line treatment in advanced HCC appeared promising for durvalumab (anti-PD-L1), pembrolizumab (anti-PD-1), and cemiplimab (anti-PD-1), while nivolumab (anti-PD-1) could be used first- or second-line. The toxicity profiles were similar to those previously described in the literature for anti-PD-1/PD-L1 in other tumor types and did not differ between molecules. No cases of HBV or HCV reactivation have been observed. Efficacy results are summarized in Table 3. However, a recent communication reported that the KEYNOTE-240 phase 3 trial, comparing pembrolizumab to placebo plus best supportive care in 413 patients previously treated with sorafenib, did not meet its co-primary endpoints of OS and PFS [49]. Indeed, despite an improvement in favor of pembrolizumab, these differences did not achieve statistical significance per the prespecified statistical plan. ORR was 16.9% for pembrolizumab vs 2.2% for placebo (*P* = 0.00001) and responses were durable (median duration of response: 13.8 months). Reasons for this failure could be previous sorafenib treatment, the progressive nature of the tumors in these patients, in whom the immune reserve was depleted and/or subsequent treatments. The KEYNOTE-394 trial is an ongoing mirror clinical trial of KEYNOTE-240 in Asian patients (NCT03062358).

In a multicohort study with durvalumab [52], the clinical benefit was greater in patients with chronic HCV infection, but this observation was limited by the small number of patients.

In a pre-specified exploratory analysis of the phase 2 study with pembrolizumab [48], the authors did not find any criteria predictive of the objective response (age, viral or non-viral etiology, AFP levels, BCLC stage, macrovascular invasion, extrahepatic metastases). Another pre-specified analysis evaluated the association between PD-L1 and the radiological response by proposing an overall expression score combining PD-L1 expression by the immune microenvironment and tumor cells (CPS score). The proposed score was defined by the number of PD-L1+ cells (≥1%) (tumor cells, lymphocytes and macrophages) divided by the total number of tumor cells. PD-L1 positivity in tumor cells did not predict radiological response, although a trend was observed (*p* = 0.08), or PFS (*p* = 0.096). In contrast, 42% of patients were positive for PD-L1 according to the CPS score and there was a significant association with ORR (32% versus 20% in CPS-positive versus negative patients, respectively, *p* = 0.021) and PFS (*p* = 0.026). Thus, considering PD-L1 expression both in the tumor and immune cell compartments improved the prediction of the response to anti-PD-1 therapy in HCC. In addition, a prospective study in 31 ICI-treated patients showed that WNT/β-catenin pathway alterations are associated with a poorer DCR (0 versus 53%) and OS (9.1 versus 15.2 months) [41]. These results support the establishment of composite scores combining PD-1 expression and molecular alterations in order to properly predict ICI response.

Finally, the CheckMate-040 phase 1/2 trial evaluated nivolumab in 3 cohorts of patients with advanced HCC: HBV-infected, HCV-infected, and non-infected, respectively [50]. This study included both treatment-naive and previously sorafenib-treated patients. No significant differences in the response according to treatment exposure and/or viral infection were observed. In line with the pembrolizumab study, PD-L1+ tumors (≥1% on tumoral cells) showed a non-significant trend for higher ORR as compared to PD-L1- tumors (26 and 19%, respectively). Several studies evaluating nivolumab in patients with advanced HCC are ongoing, including the pivotal CheckMate 459 phase 3 study comparing nivolumab with sorafenib in treatment-naive patients with advanced HCC (NCT02576509). Other studies are currently evaluating nivolumab in combination with chemoembolization (NCT03572582), radioembolization (NCT03033446), TGF-β inhibitors (NCT02423343), indoleamine dioxygenase inhibitors (NCT03695250) in patients with advanced HCC, or in neo-adjuvant and adjuvant settings (NCT03630640, NCT03383458). Other strategies may include the combination of Anti-PD-1 with cytotoxic drugs as recently reported in a trial investigating camrelizumab + FOLFOX4 or GEMOX and showing in 34 treatment naive HCC patients an ORR of 26.5% and a mPFS of 5.5 months [58]. The additive/synergistic effect of immune checkpoints inhibition and cytotoxic agents remains to be established.

#### Combination of anti-PD-1 and anti-CTLA-4

First results of immune checkpoint inhibitors combination has been recently communicated. The Checkmate-040 trial included a 3-arm randomized phase II investigating nivolumab + ipilimumab at different dose in sorafenib pretreated and Anti-PD-1 naive patients [53]. One hundred and forty-eighth number patients were randomized and 37% had high grade treatment related adverse event leading in 5% to discontinuation. Overall, the ORR was 31% with 5% (*N* = 7) of complete response. In the 3 arms the DCR ranged between 43 to 54% with higher benefit in the Nivolumab 1 mg/kg + ipilimumab 3 mg/kg (4 doses) Q3W. This arm displayed an interesting mOS of 23 months compared to the other arms (12 and 13 months). The addition of an Anti-CTLA-4 to the anti-PD-1 seems to improve the response rate but at the price of increased toxicity. Biomarker studies are pending. Current development of such combinations focuses on the neoadjuvant setting (NCT03510871, NCT03222076) or as adjuvant treatment after stereotatic radiotherapy (NCT03203304) or TACE (NCT03638141).

## Combination therapies

### Rationale for combining angiogenesis inhibitors and ICI in HCC

One of the main therapeutic goals of immuno-oncology research is to convert cold tumors into immunogenic tumors [62]. Most combination trials in HCC involved the two types of therapies that had previously shown efficacy, i.e. antiangiogenics and ICI. The rationale for this combination is based on the immunomodulatory role of VEGF-A observed in different cancers [63]. This pro-angiogenic factor is mainly produced by tumor cells, tumor-associated macrophages (TAM), and tumor-associated fibroblasts [64]. In addition, VEGF-A directly increases the recruitment of VEGFR2-expressing Treg. Moreover, a mechanism regulating T cell infiltration at the tumor-endothelium interface was recently described [65], consisting in the selective expression of Fas ligand (FasL) by the tumor endothelium (but not in the normal endothelium). This tumor-specific endothelial expression of FasL is associated with low CD8+ T cell infiltration and predominance of FoxP3+ Treg cells. VEGF-A and proinflammatory cytokines induce FasL expression by endothelial cells that acquire the ability to kill CD8+ T cells but not Treg. Pharmacological inhibition of VEGF-A leads to an increase in the number of intratumoral CD8+ cells and a reduction of tumor growth [65]. These observations highlight the critical role of VEGF-A in escaping antitumor immunity and the link between angiogenesis and immunosuppression in cancer progression. They support vascular normalization to modulate the immune microenvironment as a therapeutic approach. Consistently, several translational studies in models of non-small cell lung cancer, renal cell carcinoma or colorectal cancer have shown that anti-VEGF-A, via normalization of vascularization, increases T cell infiltration into the tumors [66–68]. The first proof-of-concept of combining anti-VEGF-A and PD-1/PD-L1 inhibitors has been brought in a renal cell carcinoma model [68], in which a combination of bevacizumab (anti-VEGF) and atezolizumab (anti-PD-L1) increased intratumoral expression of MHC class I, Th1 markers and effector T cells, leading to an increased antitumor effect.

### Results of ongoing clinical trials

Despite the absence of specific preclinical data in HCC, several trials exploring combinations of antiangiogenics and ICI are ongoing in patients with HCC. The first results of the combination of bevacizumab and atezolizumab in HCC have been reported in the IMbrave150 trial [54] showing RECIST response rates of 34% in highly selected and radiologically evaluable patients. Approximately 25% of patients showed grade 3–4 toxicity, including mostly hypertension and abnormal liver tests, but also autoimmune manifestations (e.g. diabetes, encephalitis, pneumonitis, hepatitis and pancreatitis). Despite the limited sample size, more responses were observed in patients with chronic HCV infection (43%), as previously reported, and in those with AFP ≥400 ng/mL. Importantly, 83% of responses were maintained after a median follow-up of 7.2 months. These encouraging results led to the initiation of a randomized phase 3 trial comparing atezolizumab plus bevacizumab to sorafenib in treatment-naive patients with advanced HCC [69].

Another early-phase study evaluating the association between lenvatinib and pembrolizumab has been reported [55]. Its preliminary results showed acceptable toxicity of the combination and, for the 13 evaluable patients, a radiological response rate of 46%. These encouraging results led to the initiation of a phase 3 study comparing lenvatinib to lenvatinib plus pembrolizumab in treatment-naive patients with advanced HCC (NCT03713593). In the same line of evidence, a phase Ib of axitinib+avelumab in 22 naive HCC patients has recently been communicated [57] and showed an ORR of 13.6% according to RECIST and 31.8% according to mRECIST with an acceptable safety profile.

A cohort study has been launched within the Checkmate 040 early-phase study [50] exploring the combination of ipilimumab, nivolumab, and cabozantinib. Finally, nivolumab is also being evaluated in combination with bevacizumab (NCT03382886), lenvatinib (NCT03418922) and cabozantinib (NCT03299946).

Overall, the combination of an antiangiogenic and a PD-1/PD-L1 inhibitor appears to yield better radiological response rates than each agent used as a monotherapy. The PFS results are encouraging, but safety profiles and impacts on OS have yet to be assessed in a larger population. In addition, no validated predictive biomarker is currently available to select patients who could benefit most from such strategies.

### Perspectives: tumor immune microenvironment signatures

Recent studies proposed classifications of immune microenvironment of HCCs and other tumors, mainly based on algorithms extrapolating the quantity and quality of intratumoral immune cells from messenger RNA expression of genes implicated in immune pathways.

**Fig. 2:**
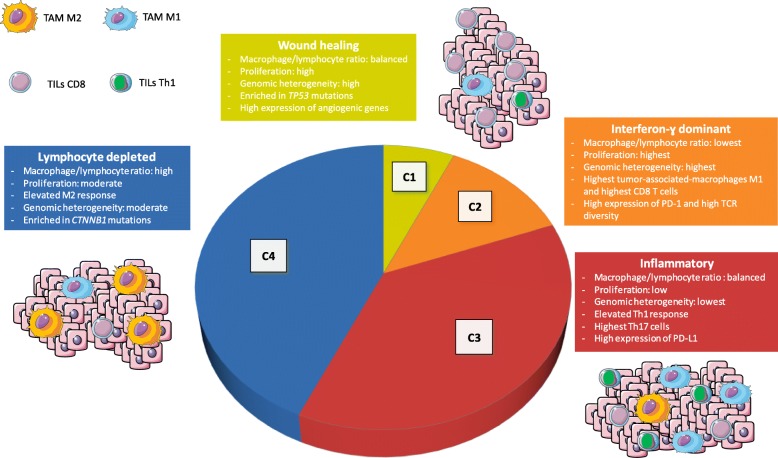
Immunological classification of hepatocellular carcinoma adapted from Thorsson et al. [70]. PD-1: programmed cell death-1; PD-L1: programmed cell death-ligand 1; TAM: tumor associated macrophage; TILs: tumor-infiltrating lymphocytes

First, a recent pan-tumor immunogenomic analysis revealed six immune contextures within tumors, which were associated with specific immune escape mechanisms [70]. The authors analyzed the distribution of the six immune patterns among HCC samples. The most frequent patterns are shown in Fig. 2. Cluster 1 (wound healing profile, 10%) and cluster 2 (interferon-γ dominant, 15%) were uncommon in HCC as compared to other types of cancers, such as breast or colorectal cancers. Interestingly, cluster 1 was associated with an elevated expression of angiogenic genes, supporting the use of angiogenesis inhibitors in these patients. Cluster 3 (inflammatory, 30%) was significantly associated with better survival as compared to other subtypes. Finally, cluster 4 (depleted in lymphocytes, 40%) was the most frequent, without significant deleterious prognostic impact. Clusters 5 and 6 (immunologically calm and TGF-β dominant, respectively), were poorly represented (< 5%) in HCC. Interestingly, the predicted neoantigen quantity was positively correlated with the amount of CD8+ T cells; high neoantigen quantities were more frequent in clusters 2 and 3, which were associated with more favorable CD8/Treg ratios than other clusters.

Further, by studying the gene expression of 66 immune markers in 196 HCC patients and using an unsupervised clustering approach, a recent analysis carried out by the TCGA consortium also uncovered the immunological heterogeneity of HCC [40]. Twenty-two percent of HCCs had significant or moderate lymphocyte infiltration, whereas 25% were poor in immune cells. Using the CIBERSORT method that predicts the immunological profile from tissue gene expression [71], the authors showed that HCC was significantly different from adjacent liver tissue in terms of immunological microenvironment, regardless of the virological status. Virally-induced tumors had the same immune profiles as virus-negative tumors (HBV+ versus HCV+, and HBV+ or HCV+ versus virus-negative, *p* > 0.05). The CD8/Treg ratio was significantly reduced in tumors, as compared to adjacent liver tissue (*p* < 1.10^− 7^), indicating a dysregulation of immune cell trafficking in the tumor. Noticeably, lymphocyte-rich tumors displayed strong expression of all 66 immunological markers, including interferon-γ (IFN-γ), and immune checkpoints such as CTLA-4 and PD-1/PD-L1.

Another recent study in a cohort of 956 HCCs reported that 25% of the tumors were rich in lymphocytes and had a high level of cytotoxic activity [72]. The authors also suggested that half of lymphocyte-rich tumors had a favorable IFN-γ pathway signature for immune checkpoint blockade activity, as already reported in melanoma or non-small cell lung cancers [73]. Some tumors also displayed a similar IFN-γ signature in the peritumoral compartment and there was no correlation between the peritumoral and the intratumoral immune profiles [72]. The other half of lymphocyte-rich tumors was characterized by exhausted immune responses and a more aggressive phenotype, in which TGF-β had a driver role by regulating metastasis, angiogenesis and epithelial-mesenchymal transition. The combination of angiogenesis and TGF-β inhibitors in this subgroup could be of interest.

**Fig. 3:**
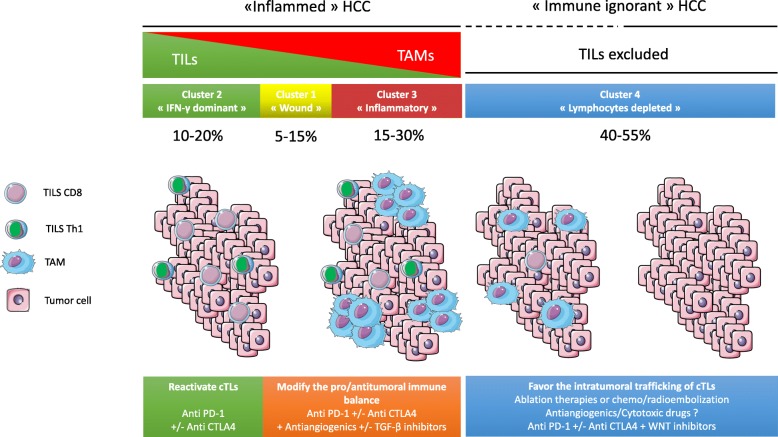
Potential combination therapies according to the immune profiles of hepatocellular carcinoma (HCC). CTLA4: cytotoxic T lymphocyte antigen-4; PD-1: programmed cell death-1; TILs: tumor-infiltrating lymphocytes; TAM: tumor-associated macrophage; TGF-β: Transforming growth factor β

Finally, WNT-β catenin pathway alterations (*CTNNB1* and *AXIN1* mutations) characterize cold tumors less responsive to immune checkpoint blockade in patients with HCC [72, 74, 75] and melanoma [76], defining primary resistance to ICI. Altogether, these results indicate that molecular alterations have a potential impact on the immune microenvironnement. Personalized immunomodulation strategies according to HCC immune profiles are proposed in Fig. 3.

Overall, half of HCC are rich in intratumoral immune cells, with different phenotypes mainly based on the amount of intratumoral lymphocytes relative to macrophages. The balance and interactions between protumoral (Treg, T-helper 17 cells, TAM M2) and antitumoral immune cells (cytotoxic CD8 cells, natural killers, TAM M1) remain to be better characterized in HCC. Moreover, the liver contains a complex immune diversity with specific populations of immune cells [51]. Liver resident macrophages, also known as Kupffer cells, is the largest population of hepatic immune cells playing with a high phenotypic plasticity, either by promoting tolerance or by promoting inflammation depending on environmental changes. Indeed, they can differentiate into M1-like macrophages releasing IL-12 and IL-23, or into M2-like macrophages depleting CD8 T cells and inducing Treg [51]. Similarly, hepatic dendritic cells can be tolerogenic by stimulating IL-27 and Treg expansion, or proinflammatory by presenting antigens to T cells [51]. Furthermore, the liver is an organ enriched in Natural Killer cells and γδ T cells whose functions are still poorly understood [56]. A better understanding of this complex immune network would help to polarize an effective anti-tumor immune response. Nevertheless, consistent with other tumors, the subgroup of HCCs with an increased IFN-γ response displays favorable immune features for next-generation immunotherapies: high expression of immune checkpoints, such as CTLA-4, PD-1, or PD-L1, high CD8+ T cell infiltration, high CD8/Treg ratio, and moderate or low TAM numbers. These observations provide a strong rationale for the use of ICI in immune cell-rich HCC.

## Conclusion

More than 70% of patients with HCC present with intermediate or advanced-stage disease (BCLC stage B, C or D) and require palliative care. Sorafenib was the first drug that demonstrated a survival benefit in patients with preserved liver function and advanced HCC. However, the OS benefit remains limited with sorafenib and it has been a long time since its approval without major therapeutic breakthrough. Thus, strategies that delay tumor progression upon first-line sorafenib therapy are currently developed, including immune checkpoints blockade and combination therapies involving antiangiogenics and ICI. Table 4 summarizes the most important ongoing clinical trials looking at immunotherapy in advanced HCC. Collaborations between clinicians and researchers to conduct innovative clinical trials including high-level translational studies may lead to the identification of biomarkers with predictive value for both clinical outcome and response to combination therapies.

**Table 4:** Summary of ongoing clinical trials of immune therapies in patients with hepatocellulars carcinoma (HCC)

*Type of immunotherapy*	*Molecules*	*NCT number*	*Phase*	*Population*	*Estimated enrollment*	*Recruitment status*
Anti-PD-1	Pembrolizumab	NCT03062358	III	Advanced HCC, pre-treated	450	Recruiting
	Nivolumab	NCT02576509	III	Advanced HCC, naive	1720	Active, not recruiting
		NCT03383458	III	Resected HCC	530	Recruiting
Combinations						
Anti-PD-1 + Anti CTLA-4	Durvalumab + tremelimumab	NCT03298451	III	Advanced HCC, naive	1310	Recruiting
	Nivolumab + ipilumumab	NCT03510871	II	Eligible for curative surgery	40	Not yet recruiting
		NCT03222076	II	Resected HCC	45	Recruiting
Angiogenesis and ICI	Nivolumab + bevacizumab	NCT03382886	I	Advanced HCC, pre-treated	12	Active, not recruiting
	Nivolumab + lenvatinib	NCT03418922	I	Advanced HCC, pre-treated and naive	30	Active, not recruiting
	Pembrolizumab + lenvatinib	NCT03713593	III	Advanced HCC, naive	750	Recruiting
Transarterial chemoembolization and ICI	Nivolumab	NCT03572582	II	Intermediate stage HCC	49	Recruiting
	Durvalumab + tremelimumab	NCT03638141	II	Intermediate stage HCC	30	Recruiting
Y90-Radioembolization and Anti-PD-1	Nivolumab	NCT03033446	II	Advanced HCC	40	Recruiting

*CTLA-4* Cytotoxic T lymphocyte-associated protein 4; *ICI* immune checkpoint inhibitors; *PD-1* programmed cell death-1; *PD-L1* Programmed death-ligand 1

## Data Availability

Not applicable.

## Abbreviations

AFP: Alpha-foeto-protein
BCLC: Barcelona-Clinic Liver Cancer
CR: Complete response
CTLA-4: Cytotoxic T lymphocyte antigen-4
DCR: Disease control rate
ECOG PS: Eastern Collaborative Oncology Group Performance Status
FasL: Fas ligand
HBV: Hepatitis B virus
HCC: Hepatocellular carcinoma
HCV: Hepatitis C virus
HR: Hazard Ratio
ICI: Immunecheckpoint inhibitors
iClust: Integrated cluster
IFN-γ: Interferon-γ
IGF: Insulin growth factor
m: Months
MKI: Multikinase inhibitor
mOS: Median overall survival
mPFS: Median progression-free-survival
mTOR: Mechanistic target of rapamycin
N: Number of randomized patients
NA: Not available
NR: Not reached
ORR: Objective response rate
OS: Overall Survival
PD-1: Programmed cell death-1
PDGF: Platelet-derived growth factor
PD-L1: Programmed cell death-ligand 1
PFS: Progression-free survival
TACE: Transarterial chemoembolization
TAM: tumor-associated-macrophages
TCGA: The Cancer Genome Atlas Research Network
TERT: Telomerase reverse transcriptase
TGF-β: Transforming growth factor β
Treg: Regulatory T lymphocytes
VEGF: Vascular endothelial growth factor
